# Physiological and clinical responses to cycling 7850 km over 85 days in a physically active middle‐aged man with idiopathic Parkinson's disease

**DOI:** 10.14814/phy2.15772

**Published:** 2023-07-20

**Authors:** Muhammad M. Kathia, Julian C. Bommarito, Avery Hinks, Elira Leake, Julia Shannon, Jenna Pitman, Barbara Connolly, Jamie F. Burr, Lori Ann Vallis, Geoffrey A. Power, Philip J. Millar

**Affiliations:** ^1^ Human Cardiovascular Physiology Laboratory, Department of Human Health and Nutritional Sciences, College of Biological Sciences University of Guelph Guelph Ontario Canada; ^2^ Neuromechanical Performance Research Laboratory, Department of Human Health and Nutritional Sciences, College of Biological Sciences University of Guelph Guelph Ontario Canada; ^3^ Gait Biomechanics Laboratory, Department of Human Health & Nutritional Sciences, College of Biological Sciences University of Guelph Guelph Ontario Canada; ^4^ Division of Neurology, Department of Medicine McMaster University Hamilton Ontario Canada; ^5^ Human Performance and Health Research Laboratory, Department of Human Health & Nutritional Sciences University of Guelph Guelph Ontario Canada

**Keywords:** cycling, exercise training, Parkinson's disease

## Abstract

This case characterizes the clinical motor, perceived fatigue, gait and balance, cardiovascular, neuromuscular, and cardiopulmonary responses after cycling 7850 km over 85 days in a physically active 57‐year‐old male with idiopathic Parkinson's disease (PD). The participant cycled 73/85 days (86%); averaging 107.5 ± 48.9 km/day over 255.4 ± 108.8 min. Average cycling heart rate was 117 ± 11 bpm. The Unified Parkinson Disease Rating Scale (UPDRS) Part III motor score decreased from 46 to 26 (−44%), while the mean Parkinson Fatigue Scale (PFS‐16) score decreased from 3.4 to 2.3 (−32%). Peak power output on a maximal aerobic exercise test increased from 326 to 357 W (+10%), while peak isotonic power of single‐leg knee extension increased from 312 to 350 W (+12%). Maximal oxygen uptake following the trip was 53.1 mL/min/kg or 151% of predicted. Resting heart rate increased from 48 to 71 bpm (+48%). The systolic and diastolic blood pressure responses to a 2‐min submaximal static handgrip exercise were near absent at baseline (∆2/∆2 mm Hg) but appeared normal post‐trip (∆17/∆9 mm Hg). Gait and static balance measures were unchanged. This case report demonstrates the capacity for physiological and clinical adaptations to a high‐volume, high‐intensity cycling regiment in a physically active middle‐aged male with PD.

## Graphical abstract

This case reports the effects of cycling 7850 km over 85 days in a physically active middle‐aged male with idiopathic Parkinson’s disease. The case cycled 73/85 days (86%); averaging 107.5 ± 48.9 km/day over 255.4 ± 108.8 min. The Unified Parkinson Disease Rating Scale Part III motor score decreased from 46 to 26 (−44%), while the mean Parkinson Fatigue Scale score decreased from 3.4 to 2.3 (−32%). Peak power output on a maximal aerobic exercise test increased from 326 to 357 W (+10%), while single‐leg knee extension peak isotonic power increased from 312 to 350 W (+12%). These findings argue against a ceiling effect of aerobic exercise training dose above guideline levels in people with Parkinson’s disease.

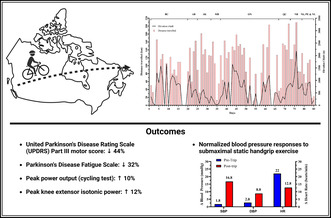

## 1 INTRODUCTION

Parkinson's disease (PD) is a progressive neurodegenerative movement disorder associated with the loss of cortical dopaminergic neurons and characterized by motor and non‐motor symptoms (Balestrino & Schapira, 2020). Patients with PD report a diminished quality of life, with impairments in physical function (Zhao et al., 2021). Aerobic and resistance exercise training are commonly recommended to maintain physical function and delay (or reverse) PD progression and symptomology (Grimes et al., 2019). However, the optimal exercise mode or dose remains unclear. Exercise intensity appears to be a key determinant, with potentially greater improvements in motor symptoms found with higher intensity (Schenkman et al., 2018) or forced exercise above voluntary capacity (Miner et al., 2020). Less clear is the role of exercise duration. Most training studies have participants exercise for ≤60 min per session (Shu et al., 2014). The effects of more prolonged exercise duration on PD symptoms remain unknown. It is also unknown whether participants already engaged in regular physical activity can achieve additional benefits with further increases in the exercise dose above current recommendations.

The purpose of this case study was to characterize adaptions in physiological and clinical parameters in a physically active middle‐aged male with idiopathic PD who cycled 7850 km over 85 days.

## 2 METHODS

### 2.1 Participant

The case was a 57‐year‐old male who was diagnosed with idiopathic PD in 2014 and prescribed 200‐mg levodopa (four times per day), 50‐mg carbidopa (four times per day), 5‐mg selegiline (two times per day), 0.75‐mg mirapex (three times per day), 200‐mg entacapone (three times per day), 100‐mg amantadine (two times per day), and 15‐mg mirtazapine (one time per day). The participant self‐reported no history of orthostatic intolerance, cardiovascular disease, diabetes, dementia, stroke, or autonomic neuropathy. He regularly engaged in aerobic exercise five times per week (~300‐min per week). Written informed consent was obtained and all protocols were approved by our institutional research ethics board in accordance with the Helsinki Declaration of 1975, as revised in 2008.

### 2.2 Experimental overview

Experimental visits were completed before and after completing his ride across Canada. The first visit was completed 3 days before the cycling trip commenced in Victoria, British Columbia, Canada. Post‐trip testing was completed 13 days after finishing in St. Johns, Newfoundland, Canada. Cycling data was captured using a Garmin Edge 830 (Garmin Ltd.) and recorded on Strava (Strava Inc.). The participant abstained from caffeine, alcohol, and strenuous physical activity for ≥12 h before each visit. Upon arriving to the lab, the participant completed balance and gait trials followed by clinical assessments. Next, cardiovascular measures were obtained at rest and during exercise. Following a 30‐min break, the participant performed a maximal cardiopulmonary exercise test on a cycle ergometer. After a 60‐min lunch break, the participant completed the neuromuscular testing protocol.

### 2.3 Gait and balance testing

Gait was evaluated using Opal inertial measurement sensors (APDM Wearable Technologies, Inc.; 128 Hz) attached to the wrists, feet, sternum, and lumbar region. Five self‐paced 13‐m walking trials were collected, with gait assessed between 2‐ to 11‐m (Kressig et al., 2006). The average over the five walking trials was calculated. Standing balance was evaluated via three 1‐min standing trials on an AMTI force plate (50 Hz) while wearing socks. Foot placement was traced on paper to ensure a consistent base of support between the pre‐ and post‐trip visits. The participant was instructed to stand quietly and look forward at a visual target on the wall. The mean value of static balance metrics (Prieto et al., 1996) were calculated for the three standing trials.

### 2.4 Clinical assessments

The participant completed the 16‐item Parkinson Fatigue Scale (PFS‐16), self‐reporting fatigue severity over the past 2 weeks (Brown et al., 2005). A trained physiotherapist assessed the participant for calculation of the Unified Parkinson Disease Rating Scale (UPDRS) Part III motor score (Goetz et al., 2008).

### 2.5 Resting cardiovascular testing

Supine resting heart rate (HR) and blood pressure (BP) were collected using an automated oscillometric device (BPTru Medical Devices). Central artery stiffness was assessed using carotid‐femoral pulse wave velocity (SphygmoCor‐XCEL, AtCor Medical Pty Ltd). HR variability (HRV) was calculated in the time (Standard deviation of interbeat intervals [SDNN] and root mean square of successive interbeat interval difference [RMSSD]) and frequency‐domain (low and high spectral power) (HRV module, ADInstruments). Cardiac baroreflex sensitivity was calculated using the sequence method, as described (Doherty et al., 2018).

### 2.6 Static handgrip exercise testing

In the supine position, the participant performed three 3–5 s maximal handgrip contractions (Model 78010, Lafayette Instrument) using his left hand, with >30 s rest between trials. After 10‐min of rest, the participant completed a 2‐min baseline and a 2‐min static handgrip contraction at 30% maximal voluntary contraction (MVC). Continuous measures of BP and HR were collected using finger photoplethysmography (Human NIBP system, ADInstruments) and reported as change from baseline to the last minute of exercise.

### 2.7 Cardiopulmonary exercise testing

Maximal exercise tests were conducted on an electronically braked cycle ergometer (Velotron, RacerMate). Following a 5‐min warm‐up, the participant started at a resistance of 100 W and increased 1‐W every 3 s until the cycling cadence could not be maintained above 50 rpm. Strong verbal encouragement was provided throughout the test. Standard cardiorespiratory measures were collected using indirect calorimetry (Quark CPET, COSMED). Data were computed in 30s rolling averages. Percent predicted VO_2_max was estimated (Jones et al., 1985).

### 2.8 Neuromuscular testing

Neuromuscular testing of the left knee extensors was performed using a HUMAC NORM dynamometer (CSMi), as described (Slysz et al., 2021). Two custom transcutaneous stimulation pads were placed on the thigh to deliver electrically evoked twitches (1000 μs, 400 V; Model DS7AH, Digitimer). Knee angle was set at 80° (full extension = 180°) for isometric MVCs and twitches, and a 80° to 140° range for isotonic ‘power’ contractions. Three minutes of rest was provided between each pre‐fatigue MVC and set of isotonic knee extensions. Neuromuscular testing proceeded in the following order: (1) peak twitch determination; (2) assessment of isometric MVC and voluntary activation (VA); (3) assessment of isotonic power; (4) the isotonic fatigue task and recovery. Current for peak twitch torque was determined by the current until the twitch torque plateaued. This current ×1.2 was subsequently used to assess VA via the interpolated twitch technique (Dalton et al., 2012). Three isometric MVC attempts were completed. For each MVC, the participant held the contraction for ~3 s and provided strong verbal encouragement and visual feedback. During the second MVC, a twitch was delivered at the MVC plateau (superimposed twitch) and ~2 s following relaxation (potentiated twitch) to calculate *%VA* (Dalton et al., 2012). A minimum of 90% VA was required before continuing. The average torque within a 500 ms window around the highest MVC's peak was taken as the MVC torque. The participant then performed two sets of three “fast and hard” knee extensions at an isotonic load of 20% MVC torque. The average peak power among the two most powerful knee extensions was taken as the peak isotonic power. For the fatigue task, the participant completed 500 isotonic knee extensions loaded at 20% MVC torque (Akagi et al., 2020). Assessment of MVC torque and VA was completed immediately, 30s, and 1‐, 2‐, 3‐, 4‐, 5‐, and 10‐min following termination of the fatigue task.

## 3 RESULTS

**FIGURE 1:**
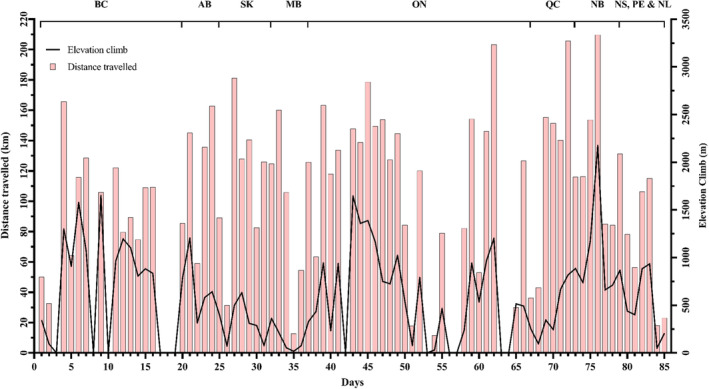
Daily distance traveled (bars) and elevation climb (line) by the participant throughout his 85‐day cycling trip across Canada. AB, Alberta; BC, British Columbia; km, kilometer; m, meter; MB, Manitoba; NB, New Brunswick; NL, Newfoundland and Labrador; NS, Nova Scotia; ON, Ontario; PE, Prince Edward Island; QC, Quebec; SK, Saskatchewan.

The participant cycled on 73 of 85 days (Figure 1), covering an average daily distance of 107.5 ± 48.9 km over 255.4 ± 108.8 min. Average cycling speed was 25.1 ± 4.6 km/h, with the average daily climb being 78.4 ± 457.4 m. A HR monitor was worn 30 of 73 days (41%), reporting an average cycling HR of 117 ± 11 beats per minute (92% heart rate reserve). Prescribed medications were unchanged throughout the trip, but the case reported frequently skipping the evening dose of levodopa and carbidopa due to improved motor symptoms. Body weight was unchanged between visits (71.7 vs. 71.9 kg).

### 3.1 Gait and balance assessments

No major changes were observed in any gait or static balance measures (Table 1).

**TABLE 1:** Balance and gait metrics before and after cycling 7850 km (mean ± SD).

Variable	Pre‐trip visit	Post‐trip visit
Clinical dynamic balance		
Mini‐BESTest (*n*/28)	28	28
Standing balance		
ML COP (cm)	0.71 ± 0.28	1.61 ± 0.18
AP COP (cm)	3.77 ± 0.34	2.40 ± 0.33
Cumulative path length (CPL; cm)	75.65 ± 7.88	129.95 ± 16.20
Average CPL velocity (cm/s)	1.26 ± 0.13	2.17 ± 0.27
ML COP velocity (cm/s)	0.01 ± 0.01	0.01 ± 0.01
AP COP velocity (cm/s)	0.01 ± 0.02	0.01 ± 0.01
Gait		
Average speed (m/s)	1.44 ± 0.02	1.37 ± 0.06
Stride length (m)	1.49 ± 0.02	1.42 ± 0.03
Step duration (s)	0.52 ± 0.01	0.52 ± 0.02
Cadence (steps/min)	116 ± 1	115 ± 3
Double support (%GC)	12.95 ± 0.74	16.09 ± 0.23
Foot strike angle (°)	29.77 ± 1.42	29.28 ± 1.12
Toe off angle (°)	38.49 ± 0.63	38.47 ± 0.86
Toe out angle (°)	2.74 ± 4.57	2.79 ± 1.46
Arm swing velocity (°/s)	275.77 ± 56.31	288.90 ± 73.40
Arm range of motion (°)	82.45 ± 13.18	75.09 ± 19.37

*Note*: Cummulative path length was calculated using totalcenter of pressure excursion in AP and ML using Pythagorean theorem for each data frame over the course of the balance trial duration; Average CPL Velocity, calculated as cumulative path length divided by trial duration; ML and AP COP velocity, calculated as absolute average instantaneous velocity. Gait average speed, calculated over five walking trials; Stride length, heel contact to heel contact of same foot; step duration, time between subsequent steps; cadence, number of steps per minute; double support, expressed as a % of gait cycle; foot strike angle, angle of foot when foot contacts ground, positive value when heel strikes ground first; Toe off angle, angle of foot when foot leaves the ground; toe out angle, lateral angle of foot when foot is in contact with the ground, positive angles indicate outward rotation; arm swing range of motion, range of arm swing; arm swing velocity, maximum rotational velocity of arm swing.

Abbreviations: AP, anterior–posterior; COP, center of pressure; CPL, cumulative path length; ML, medial‐lateral.

### 3.2 Resting cardiovascular and clinical measures

Resting HR increased from 48 to 71 bpm, while systolic (95 vs. 99 mm Hg) and diastolic (64 vs. 63 mm Hg) BP were similar between visits. Time‐domain HRV decreased post‐trip (SDNN; 65.6 vs. 43.1 ms; RMSSD; 83.5 vs. 34.8 ms), while the spectral ratio of low‐to‐high frequency power was increased (0.31 vs. 1.26) due primarily to a decrease in normalized high frequency power (54% vs. 27%). Cardiac baroreflex sensitivity was reduced (27.5 to 19.2 ms/mm Hg). Pulse wave velocity was unchanged (6.4–6.3 m/s). The UPDRS part III motor score decreased from 46 to 26, while the PFS‐16 score decreased from 3.4 to 2.3.

### 3.3 Cardiopulmonary exercise testing

Technical malfunction prevented the determination of an accurate pre‐trip VO_2_max. However, the post‐trip value was 53.1 mL/min/kg, equivalent to 151% predicted maximal oxygen consumption. Peak HR increased from 175 to 187 bpm. Peak power output increased from 326 to 357 W.

### 3.4 Cardiovascular responses to static handgrip exercise

**FIGURE 2:**
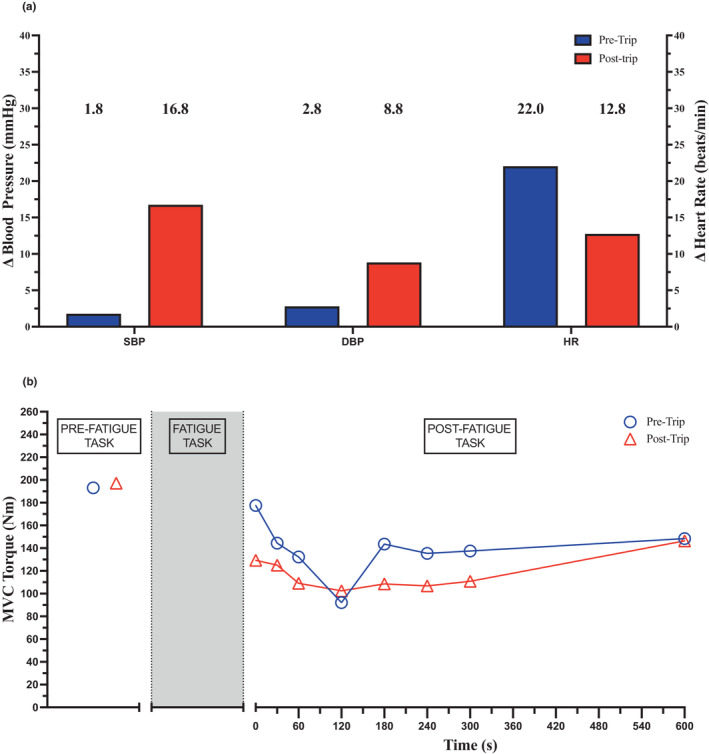
Blood pressure and heart rate response to static handgrip at 30% maximum voluntary contraction (a) and maximal voluntary contraction torque recovery following a knee extensor fatigue task (b) before and after cycling across Canada. DBP, diastolic blood pressure; HR, heart rate; N·m, Newton meter; SBP, systolic blood pressure.

Handgrip MVC was 50 kg at both visits. BP responses were increased, while the HR response was attenuated following cycling (Figure 2a).

### 3.5 Neuromuscular testing

Peak isotonic power increased ~12% from 313 to 350 W. Peak twitch torque (22.9 vs. 26.4 N m) and MVC torque (193 vs. 197 N m) increased ~15% and 2%, respectively. VA was >90% during each baseline assessment. During the post‐trip testing, the participant had a larger decrease in MVC following the fatigue task, indicating a greater ability to tolerate fatiguing exercise (Figure 2b). Recovery of VA following the fatigue task averaged 89% in pre‐testing and 86% in post‐testing.

## 4 DISCUSSION

This case demonstrates improvements in motor symptoms, perceived fatigue, peak cycling power, peak isotonic knee extensor power, and normalization of BP responses to submaximal static handgrip exercise following ~3‐months of near‐daily, high‐intensity, prolonged‐duration cycling in a 57‐year‐old physically active male with PD.

Clinically, the 20‐point reduction in the UPDRS Part III motor score is above the >10.8 threshold representing a large clinically‐important difference (Shulman et al., 2010), while the change in PFS‐16 score brought the participant below the ≥3.3 threshold associated with significant fatigue (Brown et al., 2005). The SPARX trial found that 30‐min of high‐intensity treadmill training, 3 days per week for 6 months, in participants with de novo PD had no clinically significant changes in motor symptoms but showed modest benefits compared to a usual care group (Schenkman et al., 2018). The present results are interesting given that the participant was already aerobically fit and exceeding recommended weekly exercise guidelines (i.e., >150 min per week) and thus may argue against a ceiling for which aerobic exercise may improve PD symptoms.

Despite engaging in regular cycling training, the participant increased peak power output (cycling and isotonic). Post‐trip VO_2_max was 151% of predicted values, demonstrating the capacity to maintain high cardiorespiratory fitness while living with PD for ~8 years. PD is associated with sympathetic nervous system dysregulation (Sabino‐Carvalho et al., 2021) and blunted BP responses to static handgrip exercise (Sabino‐Carvalho et al., 2018). In support, BP responses to static handgrip exercise were near‐absent pre‐trip but more closely aligned with data from healthy participants (Lee et al., 2021) at post‐testing. Whether exercise training can reverse these abnormal hemodynamic responses and reduce the risk of orthostatic hypotension, common in PD, warrants further study. Resting heart rate was higher post‐trip, accompanied by declines in HRV and cardiac baroreflex sensitivity, and suggesting reductions in tonic and reflex cardiac vagal modulation. Whether these adaptations are related to the high‐volume of high‐intensity exercise remains unknown, though it is important to note that the case displayed decreased perceived fatigue and increased peak cycling power.

Neuromuscular testing demonstrated a larger decrease in MVC torque (strength) post‐trip, which appeared to remain depressed until 10‐min of recovery. Prior cross‐sectional work reported that knee extensor fatigability is blunted in PD patients with a high UPDRS motor score (≥31.7) compared to PD patients with a low UPDRS motor score (<31.7) and healthy controls, possibly due to impaired VA causing less metabolite accumulation (Stevens‐Lapsley et al., 2012). However, our participant's UPDRS score improved from 46 to 26 after the trip. It is possible that small impairments in VA recovery following the fatigue task post‐trip were due to increased group III/IV muscle afferent sensitivity in the legs (Wan et al., 2017), which could also be involved in mediating the increased hemodynamic response to exercise. Future studies should consider the effects of exercise training on metaboreflex activation in individuals with PD.

## 5 CONCLUSIONS

This case reports improvements in motor symptoms and subjective fatigue, in conjunction with, increased peak power output, improved hemodynamic responsiveness to static handgrip exercise, and increased ability to reach a higher state of fatigue during an isotonic knee extension fatigue task following an 85 day period of high‐intensity, high duration cycling in a fit, physically active 57‐year‐old male with idiopathic PD. These observations argue against a ceiling effect of aerobic exercise training on PD motor symptoms and suggest that future clinical trials should compare the efficacy of altering exercise intensity and duration for clinical and physiological adaptations in people with PD.

## AUTHOR CONTRIBUTIONS

Muhammad M. Kathia, Jamie F. Burr, Lori Ann Vallis, Geoffrey A. Power, and Philip J. Millar, conceived and designed research; Muhammad M. Kathia, Julian C. Bommarito, Avery Hinks, Elira Leake, Julia Shannon, Jenna Pitman, and Lori Ann Vallis performed experiments; Muhammad M. Kathia, Julian C. Bommarito, Avery Hinks, Elira Leake, Julia Shannon, Lori Ann Vallis, Geoffrey A. Power, and Philip J. Millar analyzed data; Muhammad M. Kathia, Julian C. Bommarito, Avery Hinks, Elira Leake, Julia Shannon, Jenna Pitman, Barbara Connolly, Jamie F. Burr, Lori Ann Vallis, Geoffrey A. Power, and Philip J. Millar interpreted results of experiments; Muhammad M. Kathia prepared figures; Muhammad M. Kathia, Julian C. Bommarito, Avery Hinks, Elira Leake, Julia Shannon, Jenna Pitman, Lori Ann Vallis, Geoffrey A. Power, and Philip J. Millar drafted manuscript; Muhammad M. Kathia, Julian C. Bommarito, Avery Hinks, Elira Leake, Julia Shannon, Jenna Pitman, Barbara Connolly, Jamie F. Burr, Lori Ann Vallis, Geoffrey A. Power, and Philip J. Millar edited and revised manuscript; Muhammad M. Kathia, Julian C. Bommarito, Avery Hinks, Elira Leake, Julia Shannon, Jenna Pitman, Barbara Connolly, Jamie F. Burr, Lori Ann Vallis, Geoffrey A. Power, and Philip J. Millar approved final version of manuscript.

## FUNDING INFORMATION

Supported by a Parkinson Canada Pilot Project Grant (P.J.M.; 2018‐00186). P.J.M. is a recipient of an Early Researcher Award (18‐14‐288) by the Ontario Ministry of Economic Development, Job Creation and Trade.

## CONFLICT OF INTEREST STATEMENT

No conflicts of interest, financial or otherwise, are declared by the authors.

## Data Availability

The data that support the findings of this study are available from the corresponding author upon reasonable request.
